# Physician-Related Variability in the Outcomes of an Invasive Treatment for Neck and Back Pain: A Multi-Level Analysis of Data Gathered in Routine Clinical Practice

**DOI:** 10.3390/ijerph18083855

**Published:** 2021-04-07

**Authors:** Ana Royuela, Francisco M. Kovacs, Jesús Seco-Calvo, Borja M. Fernández-Félix, Víctor Abraira, Javier Zamora

**Affiliations:** 1 CIBER Epidemiología y Salud Pública (CIBERESP), 28029 Madrid, Spain; aroyuela@idiphim.org (A.R.); borjam.fernandez@hrc.es (B.M.F.-F.); javierza@gmail.com (J.Z.); 2 Unidad de Bioestadística, Hospital Puerta de Hierro Majadahonda, IDIPHISA, 28222 Madrid, Spain; 3 Spanish Back Pain Research Network, Unidad de la Espalda Kovacs, Hospital Universitario HLA-Moncloa, Avda, Valladolid, 81, 28008 Madrid, Spain; jesus.seco@unileon.es (J.S.-C.); victor.abraira@gmail.com (V.A.); 4 Kovacs Back Pain Unit, HLA-Moncloa University Hospital, 81, Avda, Valladolid, 28008 Madrid, Spain; 5 Institute of Biomedicine (IBIOMED), Campus Universitario, University of León, 24071 León, Spain; 6 Department of Physiology, University of the Basque Country, 48940 Leioa, Spain; 7 Unidad de Bioestadística Clínica, Hospital Ramón y Cajal, IRICYS, Ctra, de Colmenar Viejo km, 9100, 28034 Madrid, Spain

**Keywords:** pain, disability, neuroreflexotherapy, post-marketing surveillance, variability, learning curve

## Abstract

Neuro-reflexotherapy (NRT) is a proven effective, invasive treatment for neck and back pain. To assess physician-related variability in results, data from post-implementation surveillance of 9023 patients treated within the Spanish National Health Service by 12 physicians were analyzed. Separate multi-level logistic regression models were developed for spinal pain (SP), referred pain (RP), and disability. The models included all patient-related variables predicting response to NRT and physician-related variables. The Intraclass Correlation Coefficient (ICC) and the Median Odds Ratio (MOR) were calculated. Adjusted MOR (95% CI) was 1.70 (1.47; 2.09) for SP, 1.60 (1.38; 1.99) for RP, and 1.65 (1.42; 2.03) for disability. Adjusted ICC (95%CI) values were 0.08 (0.05; 0.15) for SP, 0.07 (0.03; 0.14) for RP, and 0.08 (0.04; 0.14) for disability. In the sensitivity analysis, in which the 6920 patients treated during the physicians’ training period were excluded, adjusted MOR was 1.38 (1.17; 1.98) for SP, 1.37 (1.12; 2.31) for RP, and 1.25 (1.09; 1.79) for disability, while ICCs were 0.03 (0.01; 0.14) for SP, 0.03 (0.00; 0.19) for RP, and 0.02 (0.00; 0.10) for disability. In conclusion, the variability in results obtained by different NRT-certified specialists is reasonable. This suggests that current training standards are appropriate.

## 1. Introduction

Subacute and chronic non-specific neck (NP) and low back pain (LBP) represent a major health, social, and economic burden [1,2,3,4,5,6,7]. Many invasive and non-invasive treatments are used for these conditions [3,6,7]. Invasive treatments require manual skills leading to physician-related variability in performance [8,9,10,11,12], even when applied by major experts on highly selected patients [13].

Neuroreflexotherapy intervention (NRT) is a minimally invasive treatment for neck and back pain [6,7,14,15,16,17,18,19,20,21,22,23]. It involves subcutaneous implantation of metallic surgical staples and epidermal burins. These penetrate the skin no more than 2 mm and remain in place for up to 90 days. The procedure intends to deactivate neurons involved in the mechanisms perpetuating pain, muscle contracture, and neurogenic inflammation [6,7,14,15,16,17,18,19,20,21,22,23]. NRT requires subtle manual skills, since precision in the location where the devices are implanted is key to efficacy [14,15,17].

NRT is one of the few treatments for neck and back pain which have been shown to lead to both statistically and clinically significant improvements in pain and disability [6,7,14,15,16,17,18,19,20,21,22,23]. It was implemented in five regions of the Spanish National Health Service (SNHS) under post-implementation surveillance, and positive clinical and economic results prompted recommendation for generalization to the entire SNHS [18,19,20,21,22,23,24,25]. Analysis of post-surveillance data made it possible to develop models that have shown to be valid for predicting individual patient response to treatment [21].

Estimating physician-related variability of results sheds light on validity of training standards, which is a pre-requisite for generalization. Therefore, the objectives of this study were to: (a) assess the variability of results obtained by different practitioners in routine practice, adjusting for patient characteristics, and (b) gain insight into the learning curve for this technology, by gathering data on the relationship between clinical results and physicians’ level of training.

## 2. Materials and Methods

All procedures followed were in accordance with the ethical standards of the Helsinki Declaration of 1975, as revised in 1983.

Participants were asked for a written informed consent, which authorized the use of demographic and clinical data deriving from their care for the purpose of this study.

This study consisted of a Clinical Audit which did not imply any changes to routine clinical practice, and data analyzed did not contain any personal data, which would allow identification of patients. Therefore, according to the Spanish law, this study did not require approval by an Institutional Review Board.

### 2.1. Setting 

This study was conducted in the Health Services of the Balearic Islands, Asturias, Cataluña, Murcia, and Madrid. These five regional Health Services belong to the Spanish National Health Service (SNHS) and cover approximately 17.6 million inhabitants, about 37.5% of the Spanish population [26].

### 2.2. Subjects

At the design phase of this study, it was decided to analyze the variability of results obtained by different practitioners once the first 9000 patients who sought care for NP or LBP within the SNHS, and had undergone NRT, had been discharged. All the patients who had been discharged after receiving NRT were included in this study, with no exclusion criteria.

Patients were treated by 12 physicians (two supervisors and 10 trainees), none of whom authored this study.

### 2.3. Intervention 

Indication criteria for NRT, are: neck, back pain, or referred pain ≥ 3 points on a 10-point visual analog scale (VAS) [27]; lasting ≥ 14 days; and not caused by systemic diseases, direct trauma or fracture. NRT is not indicated if the patient is allergic to metal, presents cauda equina syndrome, or suffers from neurogenic claudication caused by lumbar spinal stenosis [6,7,14,15,16,17,18,19,20,21,22,23]. Patients with failed back surgery can be treated. Patients with “red flags” can only be treated once the appropriate tests have ruled out that pain is caused by systemic conditions such as spondylitis, neoplastic, infectious, vascular, metabolic, visceral or endocrine-related processes [1,6,7,14,15,16,17,18,19,20,21,22,23].

**Figure 1:**
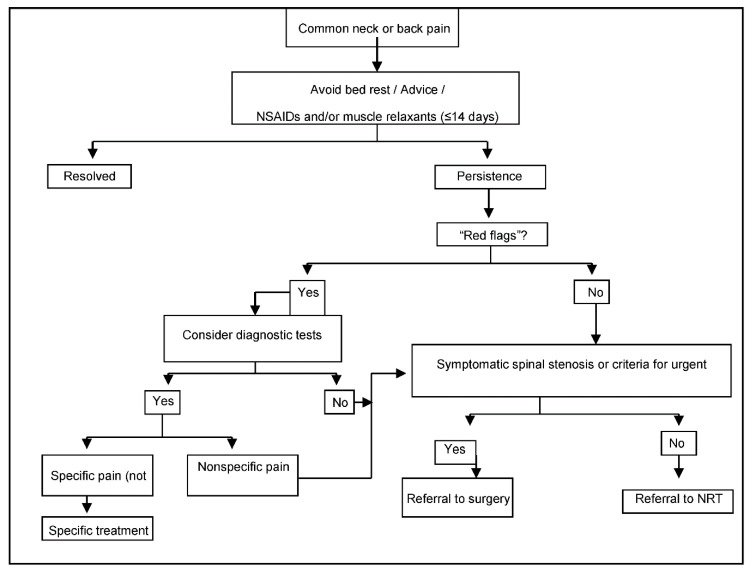
Referral protocol to NRT, within the Spanish National Health Service.

Patients are referred by primary care physicians to specialized NRT Units in each region following a standardized protocol (Figure 1). Specialists confirm indication criteria and carry out the NRT interventions after patients have signed the written consent forms, which authorize the intervention and the analysis of data gathered during post-implementation surveillance.

Twelve weeks later, the surgical material is removed and patients are discharged, unless criteria for repeating the procedure are met (pain improvement after the previous intervention ≥2 VAS points, pain severity still ≥3 VAS points, absence of relevant adverse events, and patient’s written consent) [16,17,18,19,20,21,22,23].

### 2.4. Post-Implementation Surveillance

All data analyzed in this study stem from post-implementation surveillance in routine practice. They are gathered through previously validated methods, and introduced into a database [18,19,20,21,22,23].

Data provided by the referring physician include: duration of pain (since the first episode and for the current episode, separately); reason for referral (NP, TP or LBP); date of referral; existence of referred pain (yes/no); history of spine surgery; diagnosis of failed surgery syndrome; radicular pain caused by symptomatic disc protrusion/herniation (yes/no) or symptomatic lumbar spinal stenosis (yes/no), “common” or “unspecific” syndrome (yes/no); concomitant diagnosis of fibromyalgia; other comorbidities; diagnostic tests performed (X-Rays, MRI, scanner, EMG, other); imaging findings (“disc degeneration”, “facet joint degeneration”, “scoliosis”, “difference in leg length”, “spondylolysis”, “spondylolisthesis”, “spinal stenosis”, “disc protrusion”, “disc herniation”, “other findings”, or “no findings”, according to radiologists’ reports); and treatments provided for the current episode (drugs -analgesics, NSAIDs, steroids, muscle relaxants, codeine, other opioids, other drugs-, physiotherapy/rehabilitation, surgery).

Data provided by the patient include; gender, date of birth, academic level (less than elementary school, elementary school, high school, university), current pregnancy, employment status (“working”, “not qualifying for receiving financial assistance for NP, BP or LBP”—e.g., housewife-, or “receiving financial assistance for NP, TP or LBP”—e.g., sick leave-), involvement in NP, TP or LBP-related employment claims (e.g., requesting disability pension), involvement in NP, TP or LBP-related litigation (e.g., traffic accident), and satisfaction (standardized questionnaire completed anonymously and alone by the patients).

Pain and disability are assessed at each visit to the primary care centers and the specialized NRT Units. Separate 10-cm visual analogue scales (VASs) are used for spinal pain (NP, TP or LBP) and referred pain [27]. The Roland–Morris Questionnaire (RMQ) and the Neck Disability Index (NDI) are used for LBP and NP-related disability, respectively [28,29]. Value ranges (from best to worst) are 0–10 for VAS, 0–24 for RMQ, and 0–100 for NDI [27,28,29,30,31,32]. Since the NDI is used for patients with neck pain and RMQ is used for patients with LPB, at the analysis phase, a “standardized score for disability” is calculated with a value range from 0 to 100 (from best to worst). This score reflects the percentage of the maximum possible score for neck or back pain-related disability (100 and 24 points, respectively). Data on adverse events (detected by patients, referring physicians, or NRT specialists) and use of diagnostic and other therapeutic procedures are also gathered.

### 2.5. Training Standards and Certification

Training standards for NRT imply a 3-year education program, during which 850 supervised NRT interventions per year are performed [25]. In order to qualify, trainees must match ≥95% of their supervisor’s decision on patient eligibility for treatment and obtain clinical results in line with those from certified specialists.

After certification, trainees obtain full privileges to perform NRT interventions in solo practice, and receive a code to access the software used for post-implementation surveillance. Until then, they use their supervisors’ personal code. Therefore, data from the interventions performed during training are assigned to each supervisor.

### 2.6. Analysis

Outcomes were spinal pain (SP), referred pain (RP), and disability [33]. Changes in the scores at referral and at discharge were calculated for each outcome, and patients who had experienced clinically relevant improvements were distinguished from those who had not. A “clinically relevant improvement” was defined as a score reduction in the corresponding measuring instrument, larger than the minimal clinically important change (MCIC). MCIC has been established at 30% of the baseline value, with a minimum value of 1.5 VAS points for SP and RP, 7 NDI points for NP-related disability, and 2.5 RMQ points for LBP-related disability [30,31,32,33]. These definitions made it impossible for patients with a baseline score below the cut-off point for a given variable, to show a clinically relevant improvement for that variable. Therefore, these patients were excluded from the analysis on that variable. Patients for whom the score of one of these variables at baseline or at discharge were missing were also excluded from the analysis on that variable.

Results for each outcome were analyzed by using multilevel logistic regression models [34], with patients at the first level and physicians at the second level. In the fixed-effects part of the models, the associations between variables and outcomes were appraised using the Odds Ratio (OR) with a 95 percent confidence interval (95%CI). Two models were developed for each outcome—an empty model in which only a random intercept was included, and a full model including at the patient-level all variables which have shown to predict improvement in SP, RP, and disability after NRT [21]. These variables are: gender, age (years), baseline scores for SP (VAS points), RP (VAS points) and disability (standardized disability score), reason for referral (NP or LBP), time elapsed since the first pain episode (<1 year, 1–<5 years, 5–<10 years, ≥10 years), duration of the current episode (“subacute”—14 to 89 days, “chronic”—90 to 365 days, highly chronic—>365 days) [34,35], employment status (“passive”, or “working”), type of pain (“radicular pain caused by symptomatic disc protrusion/herniation or lumbar spinal stenosis” vs. “common NP or LBP”), diagnosis of fibromyalgia, other comorbidities, involvement in employment claims, diagnostic tests undertaken until referral to NRT (X-rays, MRI, other), imaging findings, history of spine surgery, and treatments used prior to referral for NRT). At the physician level, the model included the number of years each physician had been performing NRT interventions in solo practice (i.e., after having been certified).

Data from patients treated for thoracic pain were excluded, since predictive models have only been developed for NP and LBP [21]. Only the first pain episode was analyzed for each patient because the number of pain episodes per patient was low, and, when combining “patient” and “physician” in one single level, very few replicates were available to assess variability.

Empirical Bayes’ residuals were calculated for each physician [36]. In each model, the variability at the “physician” level was estimated through the Median Odds Ratio (MOR) and the Intraclass Correlation Coefficient (ICC). The latter was adapted to logistic regression by the latent variable method [37,38].

The ICC quantifies the fraction of the total variability in outcomes which is attributable to the physicians. ICC values range from 0 to 1. The higher the ICC value, the greater the variability that can be attributed to physicians. For instance, an ICC = 0.06 shows that 6% of the total individual differences over the odds of improvement occurs at the physician level and might be attributable to contextual physician factors.

Conceptually, MOR reflects the degree of variability in clinical results which stem from the fact that the patients are treated by different physicians. A higher MOR reflects a higher variability. The MOR quantifies the difference in results obtained by different physicians, by comparing clinical results from two patients treated by two different physicians selected randomly. For instance, considering two patients with the same covariates, selected randomly among cases treated by two different physicians, the MOR is the median odds ratio between the patient with higher odds and the patient with lower odds [38], and can be interpreted as the median increased odds of improvement, if a given patient was treated by another better performing physician. A MOR value of 3 would mean that the median odds of a patient improving if treated by the physicians obtaining the best results is 3 times higher than the odds for improving if treated by the physicians obtaining the worst results. In this study, the MOR shows the extent to which the probability that an individual patient will improve (in terms of pain severity and disability) is determined by the treating physician [39].

A sensitivity analysis was performed, in which the models were repeated restricting data to patients treated by the trainees after they were certified as NRT specialists (i.e., data on interventions assigned to codes corresponding to supervisors were eliminated).

Stata version 13.0 software was used for statistical analysis (StataCorp. 2013. Stata Statistical Software. College Station, TX, USA: StataCorp LP).

## 2.7. Role of the Funding Source and Conflicts of Interest-Associated Biases

This study was promoted by the Spanish Back Pain Research Network, a not for profit research Organization with no links to the health industry. No external funds were received to fund this study. Only the authors were responsible for the design and conduction of the study; data collection, management, analysis and interpretation; preparation, review and approval of the manuscript; or the decision to submit the article for publication. None of the authors received any payment for this work, and none harbor any conflicts of interest.

## 3. Results

**Figure 2:**
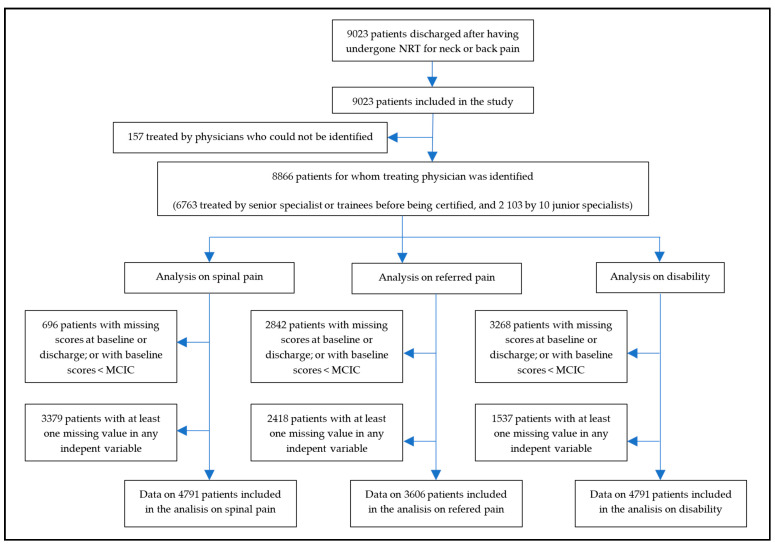
Flow chart of the study.

Since post-marketing surveillance was applied simultaneously at centers in different geographic locations, when recruitment for this study stopped, 9023 patients had been discharged after having undergone NRT for neck or back pain. Therefore, all 9023 patients were included in this study. There were no losses to follow-up. Figure 2 shows the flow chart of the study.

The identity of the treating physician was missing for 157 (1.8%) patients. Among the 12 physicians who were identified, the median (P25; P75) number of patients treated in solo practice was 180 (44; 649). The number of NRT interventions performed by, or assigned to, the two supervisors was 6763. Two trainees never qualified and are not identified in Table 1; data on the evolution of the 115 patients they had treated appear assigned to their supervisors. The sensitivity analysis included data on the 2103 patients treated by the 10 junior specialists after they were certified (Table 1).

The proportion of patients who presented missing scores for spinal pain, referred pain or disability at baseline or discharge, or had a baseline score below the corresponding MCIC, were 8%, 32%, and 37%, respectively. Additionally, patients with missing completely at random (MCAR) scores for any of the independent variables, which were introduced in the full models, were 3379 for the model on spinal pain, 2418 for the one on referred pain, and 1537 for the one on the disability. Therefore, the model on spinal pain included data from 4791 patients, the one on referred pain included data from 3606 patients, and the one on disability included data from 4061 patients (Figure 2).

Clinical results obtained by each physician are shown in Table 1, while Table 2 shows the distribution of physician and patient characteristics across the patients who did and did not show clinically relevant improvements in SP, RP, and disability after NRT.

Adjusted MOR (95%IC) was 1.70 (1.47; 2.09) for SP, 1.60 (1.38; 1.99) for RP, and 1.65 (1.42; 2.03) for disability. Adjusted ICC (95%IC) values were 0.08 (0.05; 0.15) for SP, 0.07 (0.03; 0.14) for RP, and 0.08 (0.04; 0.14) for disability. In the sensitivity analysis, adjusted MOR was 1.38 (1.17; 1.98) for SP, 1.37 (1.12; 2.31) for RP, and 1.25 (1.09; 1.79) for disability, while ICCs were 0.03 (0.01; 0.14) for SP, 0.03 (0.00; 0.19) for RP, and 0.02 (0.00; 0.10) for disability (Table 3, Table 4 and Table 5).

**Figure 3:**
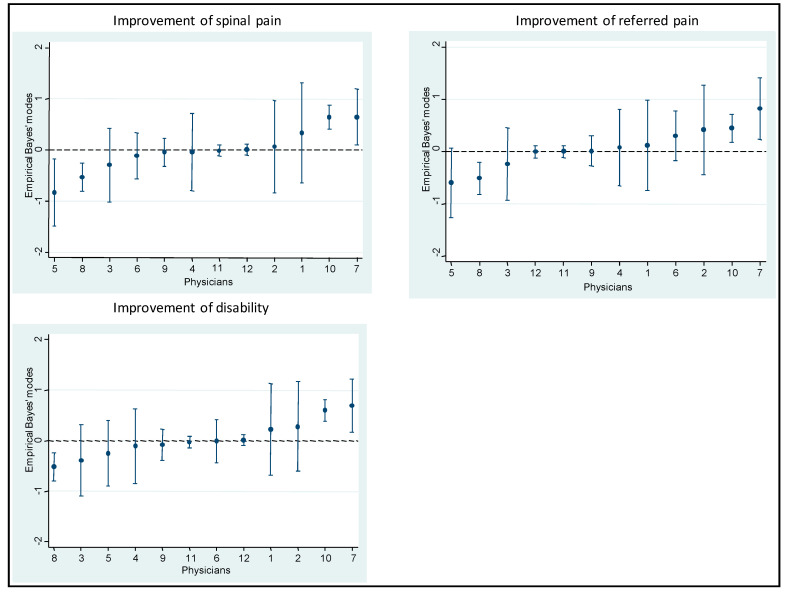
Empirical Bayes’ residuals of the variability of each physician for each outcome.

**Figure 4:**
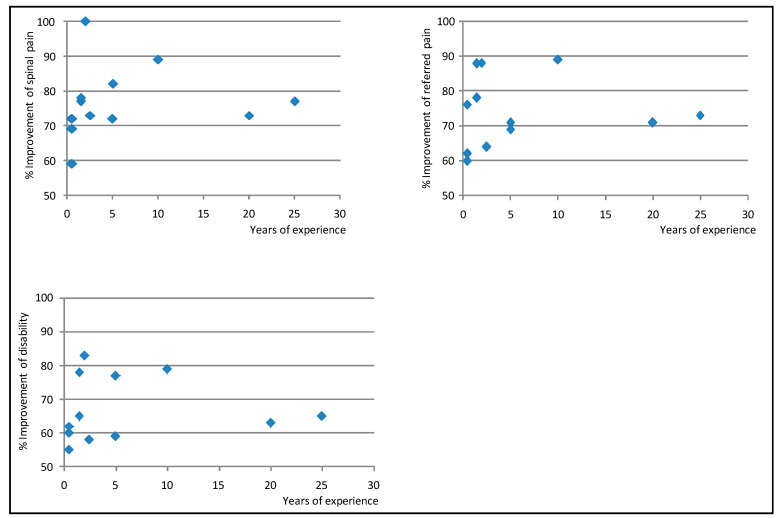
Years of experience for each physician, and improvement in neck and back pain, referred pain, and disability *. *: Note that the figure shows improvement in pain and disability between 50% and 100% of baseline values (not between 0 and 100%). Therefore, differences across physicians appear to be larger than they are.

Table 6, Table 7 and Table 8 show results from the multilevel models on the strength of the association between predictors and improvement in spinal pain, referred pain, and disability. The proportion of patients who improved and did not improve was virtually the same in both the empty and the full models for these variables (Table 9). Figure 3 shows the Empirical Bayes’ residuals of each physician’s variability for each outcome, and Figure 4 shows the number of years of experience for each physician, and the improvement in outcomes.

## 4. Discussion

Previous studies have shown that NRT improves pain and disability in 84–89% of subacute and chronic neck and back patients, and that this improvement is clinically relevant in 72–76% patients [18,19,20,21,22,23]. Results from the current study suggest that, after having adjusted for patient-related characteristics which predict individual responses to NRT, changing the physician who performs the procedure is associated with a variation of 60–70% in the odds of experiencing a clinically relevant improvement (MOR values for SP, RP and disability 1.70, 1.60, and 1.65, respectively), and that the physician who performs NRT accounts for 7–8% of the variability in patients’ evolution (ICC values for SP, RP, and disability 0.08, 0.07, and 0.08, respectively) (Table 3, Table 4 and Table 5). These figures decrease to 25–38% and 2–3% respectively, when the analysis is restricted to results obtained by certified specialists (Table 3, Table 4 and Table 5).

Several factors can account for this reduction. In the complete dataset, the number of observations was higher and the number of years in practice and the number of patients assigned to each physician were skewed. Moreover, in the analysis of the complete data set, results from the interventions for which the worst and best results were to be expected (i.e., those performed by trainees at the beginning of their training period, and by senior experts) were identified with the same codes, which impeded analyzing them separately. All of the above may have contributed to the MOR and ICC values being larger in this analysis than in the analysis in which only data from certified specialists were included.

Senior experts who participated in this study can be seen as those with the maximum level of competency in performing NRT interventions; they had ≥20 years of experience and had shown to obtain positive clinical results in RCTs and studies conducted in routine practice [14,15,18,19,22]. However, results obtained by recently certified specialists were better than the combined results of these senior experts and the same junior specialists during their 3-year pre-certification training (Table 1 and Table 6, Table 7, Table 8, and Figure 4). At the end of the training period, all physicians obtained improvements in pain ≥60% of baseline value, which is unusually positive for patients with subacute and chronic neck and back pain treated in routine clinical practice. Some physicians obtained better results sooner than others, but, in general, between 3 and 5 years after certification, results across physicians became similar (Figure 4). This suggests that training sharply increases the proficiency of trainees, but that the learning curve for this procedure is long. In fact, the number and specific location of the surgical devices implanted in a NRT intervention are determined through physical examination and subtle manual palpation, vary from one patient to another, and are essential for the procedure to be effective; the insertion of the same number of devices within a 5 cm-radius of the target zone has consistently been shown to have virtually no clinical effect [14,15,17,40].

Whether a shorter or less intensive training would be enough is unknown and could be explored in future studies. Nevertheless, this study reflects that current training standards are effective at ensuring that certified specialists obtain results which are similar to those obtained by senior specialists (Table 1 and Table 6, Table 7, Table 8, and Figure 4) [14,15,16,17,18,19,20,21,22,23]. This suggests that, when the recommendation to roll out NRT to other geographical settings is considered [6,16,18,19,20,21,22,23], the current training standards will be adequate.

Concerns have been expressed about the feasibility of accurately assessing the clinical performance of physicians, especially for procedures requiring manual skills, given that awareness that their results are being monitored might result in changes in behavior or in avoiding treating patients they feel might have a worse prognosis [41,42]. However, these concerns are not likely to challenge the results of this study, since post-marketing surveillance mechanisms have been in force uninterruptedly since NRT was first implemented in the SNHS and include all patients who have undergone this procedure. Furthermore, physicians treating the more complex cases have no reason to fear that this could penalize data on their performance, since they are aware that results are adjusted by the individual prognosis of each patient [21].

Most reports on learning curves or variability across practitioners for invasive treatments for neck or low back pain focus on interventions other than NRT, many of which lack high quality evidence supporting their efficacy and effectiveness. Moreover, they are based on small samples (e.g., ≤150 patients or ≤3 practitioners), do not use multi-level analyses to adjust results for patient prognosis as established by previously validated models, or have been conducted either retrospectively or outside routine practice conditions (e.g., based on data gathered in randomized controlled trials) [8,9,10,11,12,13,40,41,43,44,45,46,47,48,49,50,51,52,53,54]. Therefore, it is inappropriate to compare such data with results from this study.

To date, although some invasive procedures used for neck or back pain have been assessed [55,56,57], most lack solid evidence on their efficacy or effectiveness [6,7,58,59], and very few undergo post-marketing surveillance, which makes it difficult to establish any reliable benchmarks. If, as suggested [60,61], in the future, all invasive health technologies are subject to a more stringent assessment and surveillance processes, and it will be possible to compare results from this study with data on other procedures. It is also difficult to compare physician-related variability in results when using NRT to the one obtained when using other invasive procedures, since very few other invasive treatments for neck and back pain are subject to systematic and validated post-implementation surveillance mechanisms in routine practice [18,19,20,21,22,23,24,25]. Generalizing such mechanisms to all treatments for neck and back pain will make it possible to compare physician-related variability across treatments.

Data analyzed in this study stem from surveillance mechanisms in routine practice. These mechanisms encompass all patients who undergo NRT within the Spanish National Health Service, with very little losses to follow-up, and use previously validated methods [18,19,20,21,22,23]. Therefore, validity of data is not a major concern. However, this study has several weaknesses. It was impossible to distinguish the results obtained by the trainees during their training period from those obtained by the senior experts who acted as their supervisors. In the future, trainees will be assigned a personal code throughout their entire traineeship period, which will make it possible to analyze the results they obtain separately. Missing data impeded inclusion of data from all patients in the full models on spinal pain, referred pain, and disability (Figure 2). However, it is unlikely that missing data introduced any biases, since the proportion of patients who improved and did not improve were virtually the same in the empty and full models (Table 9). The number of years of solo practice after certification and the number of patients treated by each physician were skewed; this may influence the stability of results from the models. This is due to the fact that, in order to comply with the training standards, new trainees are only recruited when the procedure is implemented in a new territory. Generalizing this procedure will make it possible to resolve this.

## 5. Conclusions

In conclusion, results from this study suggest that, at the end of their training period, NRT-certified specialists achieve clinical results, which, after adjusting for patient characteristics, are reasonably similar, which suggests that current training standards are valid for generalizing this technology.

## Figures and Tables

**Table 1:** Results obtained by each physician.

Physician	Years of Experience	Number of Patients	Spinal (Neck or Low Back) Pain (*n* = 4791)		Referred Pain (*n* = 3606)		Disability (*n* = 4061)	
			Showed a Clinically Relevant Improvement (*n* = 3548)	Did Not Show a Clinically Relevant Improvement (*n* = 1243)	Showed a Clinically Relevant Improvement (*n* = 2530)	Did Not Show a Clinically Relevant Improvement (*n* = 1076)	Showed a Clinically Relevant Improvement (*n* = 2661)	Did Not Show a Clinically Relevant Improvement (*n* = 1400)
1	2	10	10 (100)	0 (0.0)	7 (87.5)	1 (12.5)	5 (83.3)	1 (16.7)
2	1.5	13	10 (76.9)	3 (23.1)	7 (87.5)	1 (12.5)	7 (77.8)	2 (22.2)
3	0.5	38	18 (69.2)	8 (30.8)	13 (61.9)	8 (38.1)	12 (54.5)	10 (45.5)
4	1.5	42	29 (78.4)	8 (21.6)	25 (78.1)	7 (21.9)	11 (64.7)	6 (35.3)
5	0.5	48	24 (58.5)	17 (41.5)	18 (60.0)	12 (40.0)	21 (60.0)	14 (40.0)
6	0.5	167	103 (72.0)	40 (28.0)	89 (76.1)	28 (23.9)	63 (61.8)	39 (38.2)
7	10	170	137 (89.0)	17 (11.0)	100 (88.5)	13 (11.5)	80 (79.2)	21 (20.8)
8	2.5	396	240 (73.2)	88 (26.8)	136 (63.9)	77 (36.1)	137 (57.6)	101 (42.4)
9	5	578	261 (71.7)	103 (28.3)	201 (68.8)	91 (31.2)	126 (58.9)	88 (41.1)
10	5	647	501 (81.5)	114 (18.5)	226 (71.3)	91 (28.7)	431 (76.7)	131 (23.3)
11 *	20	3153	2066 (72.7)	776 (27.3)	1610 (70.7)	667 (29.3)	1048 (63.4)	604 (36.6)
12 *	25	3604	2453 (76.8)	740 (23.2)	1673 (73.4)	607 (26.6)	1513 (65.0)	814 (35.0)

*: Senior experts acting as trainee supervisors. NRT interventions attributed to them include those performed by trainees during the pre-certification, training period.

**Table 2:** Characteristics of patients who showed and did not show clinically relevant improvements in SP, RP, and disability, after NRT.

Variables	Spinal Pain (*n* = 4791)		Referred Pain (*n* = 3606)		Disability (*n* = 4061)	
	Improved (*n* = 3548)	Did Not Improve (*n* = 1243)	Improved (*n* = 2530)	Did Not Improve (*n* = 1076)	Improved (*n* = 2661)	Did Not Improve (*n* = 1400)
**Patient level**						
Gender (male) *	2384 (67.2)	806 (64.8)	1716 (67.8)	739 (68.7)	1741 (65.4)	945 (67.5)
Age (years) ^¥^	53 (42; 64)	52 (42; 65)	53 (42; 64)	53 (44; 65)	52 (41; 64)	54 (43; 65)
Reason for referral to NRT *						
Neck Pain	646 (18.2)	212 (17.1)	464 (18.3)	176 (16.4)	301 (11.3)	203 (14.5)
Low Back Pain	2902 (81.8)	1031 (82.9)	2066 (81.7)	900 (83.6)	2360 (88.7)	1197 (85.5)
Type of pain *						
Non-specific	3391 (95.6)	1162 (93.5)	2352 (93.0)	1002 (93.1)	2534 (95.2)	1308 (93.4)
Radicular pain caused by disc protrusion/herniation or spinal stenosis	157 (4.4)	81 (6.5)	178 (7.0)	74 (6.9)	127 (4.8)	92 (6.6)
Employment status *						
Not working	1440 (40.6)	506 (40.7)	1029 (40.7)	464 (43.1)	1000 (37.6)	612 (43.7)
Currently working	2108 (59.4)	737 (59.3)	1501 (59.3)	612 (56.9)	1661 (62.4)	788 (56.3)
Duration of the pain since diagnostic categorized *						
≤1 year	555 (15.6)	167 (13.4)	377 (14.9)	145 (13.5)	437 (16.4)	178 (12.7)
1–5 years	1077 (30.4)	410 (33.0)	774 (30.6)	347 (32.2)	821 (30.9)	445 (31.8)
5–10 years	909 (25.6)	327 (26.3)	669 (26.4)	286 (26.6)	665 (25.0)	366 (26.1)
>10 years	1007 (28.4)	339 (27.3)	710 (28.1)	298 (27.7)	738 (27.7)	411 (29.4)
Duration of the pain episode (days) categorized *	1022 (28.8)	235 (18.9)	706 (27.9)	193 (17.9)	810 (30.4)	290 (20.7)
Subacute (≤90 days)	1601 (45.1)	614 (49.4)	1147 (45.3)	541 (50.3)	1174 (44.1)	687 (49.1)
Chronic (91–365 days)	925 (26.1)	394 (31.7)	677 (26.8)	342 (31.8)	677 (25.4)	423 (30.2
Highly chronic (>365 days)	124 (3.5)	42 (3.4)	90 (3.6)	43 (4.0)	84 (3.2)	50 (3.6)
Diagnosis of fibromyalgia *	1471 (41.5)	590 (47.5)	1044 (41.3)	528 (49.1)	1028 (38.6)	662 (47.3)
Other comorbidities *	16 (0.5)	19 (1.5)	16 (0.6)	15 (1.4)	14 (0.5)	17 (1.2)
Involved in work-related claims *	7 (6; 8)	7 (6; 8)	7 (6; 9)	7 (6; 8)	7 (5; 8)	7 (6; 8)
Baseline severity of SP (VAS) ^¥^	7 (5; 8)	7 (5; 8)	7 (5; 8)	7 (5; 8)	6.5 (5; 8)	7 (5; 8)
Baseline severity of RP (VAS) ^¥^	54.0 (37.5; 70.8)	58.3 (41.7; 75.0)	54.2 (37.5; 70.8)	62 (42; 75)	54.2 (37.5; 70.8)	54.2 (37.5; 70.8)
Baseline disability (standardized 0–100 score) ^¥^						
Previous lumbar surgery * (yes)	258 (7.3)	169 (13.6)	205 (8.1)	151 (14.0)	196 (7.4)	184 (13.1)
Failed back syndrome * (yes)	33 (0.9)	26 (2.1)	27 (1.1)	17 (1.6)	24 (0.9)	29 (2.1)
Diagnostic procedures during the episode *						
X-ray	907 (25.6)	257 (20.7)	615 (24.3)	219 (20.4)	674 (25.3)	304 (21.7)
MRI	1189 (33.5)	410 (33.0)	838 (33.1)	387 (36.0)	940 (35.3)	459 (32.8)
Other ^¤^	230 (6.5)	84 (6.8)	174 (6.9)	76 (7.1)	173 (6.5)	95 (6.8)
Imaging findings *						
Disc degeneration	1699 (47.9)	642 (51.6)	1187 (46.9)	588 (54.6)	1188 (44.6)	746 (53.3)
Facet joint degeneration	404 (11.4)	147 (11.8)	281 (11.1)	136 (12.6)	283 (10.6)	189 (13.5)
Scoliosis	220 (6.2)	69 (5.6)	142 (5.6)	58 (5.4)	157 (5.9)	89 (6.4)
Spondylolisis	70 (2.0)	20 (1.6)	44 (1.7)	21 (2.0)	55 (2.1)	27 (1.9)
Spondylolisthesis	149 (4.2)	56 (4.5)	98 (3.9)	52 (4.8)	98 (3.7)	69 (4.9)
Spinal stenosis	189 (5.3)	95 (7.6)	149 (5.9)	96 (8.9)	131 (4.9)	106 (7.6)
Disc protrusion or herniation (extrusion)	1110 (31.3)	459 (36.9)	820 (32.4)	417 (38.8)	839 (31.5)	484 (34.6)
Other non-relevant findings ^∞^	793 (22.4)	265 (21.3)	531 (21.0)	240 (22.3)	499 (18.8)	298 (21.3)
No findings	1312 (37.0)	428 (34.4)	963 (38.1)	361 (33.6)	1065 (40.0)	463 (33.1)
Treatments						
Drugs *						
Analgesics	2347 (66.1)	816 (65.6)	1696 (67.0)	730 (67.8)	1750 (65.8)	957 (68.4)
NSAIDs	2298 (64.8)	750 (60.3)	1643 (64.9)	665 (61.8)	1746 (65.6)	898 (64.1)
Steroids	213 (6.0)	77 (6.2)	166 (6.6)	81 (7.5)	166 (6.2)	90 (6.4)
Muscle relaxants	776 (21.9)	243 (19.5)	571 (22.6)	218 (20.3)	612 (23.0)	287 (20.5)
Opioids	118 (3.3)	68 (5.5)	86 (3.4)	53 (4.9)	96 (3.6)	78 (5.6)
Other	893 (25.2)	330 (26.5)	605 (23.9)	303 (28.2)	682 (25.6)	393 (28.1)
Non pharmacological treatments *						
Physical therapy/Rehabilitation	398 (11.2)	147 (11.8)	303 (12.0)	128 (11.9)	279 (10.5)	167 (11.9)
**Physician level**						
Years of experience ^¥^	20 (8; 25)	20 (20; 25)	20 (20; 25)	20 (20; 25)	20 (10; 25)	20 (20; 25)

* Frequency (%); ^¥^ Median (P25; P75); Type of pain: “Radicular pain caused by disc protrusion/herniation or spinal stenosis” if; (a) Severity of referred pain ≥ local pain, (b) corresponding imaging finding on MRI, (c) distribution of pain consistent with the nerve root compressed by the corresponding imaging finding. “Non-specific pain”, if one or more of these criteria were not met.; ¤ Other diagnostic procedures: EMG, CT scan and other; ∞ Other imaging findings: annular tear, loss of cervical lordosis, loss of thoracic cifosis, loss of lumbar lordosis, horizontalization of the sacrum, lumbarization of S1, sacralization of L5; NRT: Neuroreflexotherapy intervention; SP: Severity of spinal pain; RP: Severity of referred pain; VAS: Visual Analog Scale (range from better to worse; 0–10).

**Table 3:** Estimates of the inter-physician variability for improvement in spinal pain (SP).

**All the Patients (*n* = 4791) †**		
	**Empty Model**	**Full Model**
Intra-class correlation coefficient (ICC) (95% CI)	0.07 (0.03; 0.14)	0.08 (0.05; 0.15)
MOR (95% CI)	1.59 (1.37; 2.00)	1.70 (1.47; 2.09)
**Sensitivity Analysis (*n* = 1230) £**		
	**Empty Model**	**Full Model**
Intra-class correlation coefficient (ICC) (95% CI)	0.03 (0.01; 0.14)	0.03 (0.01; 0.14)
MOR (95% CI)	1.36 (1.14; 2.04)	1.38 (1.17; 1.98)

† Only includes patients whose spinal pain at baseline was higher than the minimal clinically important change, and for whom data on this variable at baseline and discharge were available. £ Restricted to patients treated by physicians after the latter became certified NRT practitioners.

**Table 4:** Estimates of the inter-physician variability for improvement in referred pain (RP).

**All the Patients (*n* = 3606) †**		
	**Empty Model**	**Full Model**
Intra-class correlation coefficient (ICC) (95% CI)	0.04 (0.01; 0.16)	0.07 (0.03; 0.14)
MOR (95% CI)	1.43 (1.19; 2.13)	1.60 (1.38; 1.99)
**Sensitivity Analysis (*n* = 832) £**		
	**Empty Model**	**Full Model**
Intra-class correlation coefficient (ICC) (95% CI)	0.07 (0.01; 0.27)	0.03 (0.00; 0.19)
MOR (95% CI)	1.60 (1.23; 2.84)	1.37 (1.12; 2.31)

† Only includes patients whose referred pain at baseline was higher than the minimal clinically important change, and for whom data on this variable at baseline and discharge were available. £ Restricted to patients treated by physicians after the latter became certified NRT practitioners.

**Table 5:** Estimates of the inter-physician variability for improvement in disability.

**All the Patients (*n* = 4061) †**		
	**Empty Model**	**Full Model**
Intra-class correlation coefficient (ICC) (95% CI)	0.02 (0.01; 0.05)	0.08 (0.04; 0.14)
MOR (95% CI)	1.30 (1.20; 1.48)	1.65 (1.42; 2.03)
**Sensitivity Analysis (*n* = 1039) £**		
	**Empty Model**	**Full Model**
Intra-class correlation coefficient (ICC) (95% CI)	0.04 (0.01; 0.15)	0.02 (0.00; 0.10)
MOR (95% CI)	1.45 (1.21; 2.06)	1.25 (1.09; 1.79)

† Only includes patients whose disability at baseline was higher than the minimal clinically important change, and for whom data on this variable at baseline and discharge were available. £ Restricted to patients treated by physicians after the latter became certified NRT practitioners.

**Table 6:** Multilevel full model to determine the strength of the association between predictors and improvement in spinal pain (SP) (*n* = 4791).

In-Patient Improvement in SP	Full Model
	OR (95% CI)
Predictor	
**Patient level**	
Gender (male)	1.09 (0.94; 1.26)
Age (years)	1.00 (0.99; 1.00)
Reason for referral to NRT	
Neck Pain	Ref. cat.
Low Back Pain	1.12 (0.92; 1.38)
Type of pain	
Non-specific	Ref. cat.
Radicular pain *	1.12 (0.81; 1.55)
Employment status	
Not working	Ref. cat.
Currently working	0.92 (0.78; 1.09)
Duration of the pain since diagnostic categorized	
≤1 year	Ref. cat.
1–5 years	0.86 (0.69; 1.07)
5–10 years	0.94 (0.75; 1.18)
>10 years	1.03 (0.82; 1.30)
Duration of the pain episode (days) categorized	
Subacute (≤90 days)	Ref. cat.
Chronic (91–365 days)	0.64 (0.54; 0.76)
Highly chronic (>365 days)	0.56 (0.46; 0.68)
Diagnosis of fibromyalgia (yes)	1.10 (0.76; 1.61)
Other comorbidities	0.79 (0.65; 0.95)
Involved in work-related claims	0.39 (0.19; 0.78)
Baseline severity of SP (VAS)	1.14 (1.09; 1.19)
Baseline severity of RP (VAS)	0.94 (0.92; 0.96)
Baseline disability (standardized 0–100 score)	0.99 (0.98; 0.99)
Previous lumbar surgery (yes)	0.60 (0.48; 0.76)
Failed back syndrome (yes)	0.56 (0.31; 1.01)
Diagnostic procedures during the episode	
X-ray	1.10 (0.93; 1.30)
MRI	1.06 (0.90; 1.25)
Other	0.99 (0.74; 1.30)
Imaging findings	
Disc degeneration	0.84 (0.67; 1.05)
Facet joint degeneration	1.11 (0.87; 1.40)
Scoliosis	0.98 (0.73; 1.33)
Spondylolisis	1.15 (0.67; 1.96)
Spondylolisthesis	0.92 (0.66; 1.30)
Spinal stenosis	0.82 (0.62; 1.10)
Disc protrusion or herniation (extrusion)	0.72 (0.59; 0.87)
Other non-relevant findings	1.06 (0.87; 1.29)
No findings	0.75 (0.57; 0.99)
Treatments	
Drugs	
Analgesics	0.95 (0.79; 1.14)
NSAIDs	1.28 (1.07; 1.52)
Steroids	0.95 (0.71; 1.28)
Muscle relaxants	1.09 (0.91; 1.31)
Opioids	0.60 (0.43; 0.85)
Other	0.92 (0.77; 1.10)
Non pharmacological treatments	
Physical therapy/Rehabilitation	0.89 (0.72; 1.11)
Years of experience	0.98 (0.97; 0.99)

* Radicular pain caused by disc protrusion/herniation or spinal stenosis.

**Table 7:** Multilevel full model to determine the strength of the association between predictors and improvement in referred pain (RP) (*n* = 3606).

In-Patient Improvement in RP	Full Model
	OR (95% CI)
Predictor	
**Patient level**	
Gender (male)	0.91 (0.77; 1.08)
Age (years)	1.00 (0.99; 1.01)
Reason for referral to NRT	
Neck Pain	Ref. cat.
Low Back Pain	1.04 (0.83; 1.30)
Type of pain	
Non-specific	Ref. cat.
Radicular pain *	0.96 (0.68; 1.36)
Employment status	
Not working	Ref. cat.
Currently working	0.98 (0.81; 1.18)
Duration of the pain since diagnostic categorized	
≤1 year	Ref. cat.
1–5 years	0.96 (0.75; 1.22)
5–10 years	1.07 (0.83; 1.38)
>10 years	1.18 (0.91; 1.52)
Duration of the pain episode (days) categorized	
Subacute (≤90 days)	Ref. cat.
Chronic (91–365 days)	0.62 (0.51; 0.76)
Highly chronic (>365 days)	0.56 (0.45; 0.70)
Diagnosis of fibromyalgia	1.02 (0.69; 1.51)
Other comorbidities	0.84 (0.68; 1.03)
Involved in work-related claims	0.59 (0.28; 1.24)
Baseline severity of SP (VAS)	0.95 (0.90; 1.00)
Baseline severity of RP (VAS)	1.12 (1.07; 1.17)
Baseline disability (standardized 0–100 score)	0.99 (0.98; 0.99)
Previous lumbar surgery (yes)	0.56 (0.43; 0.72)
Failed back syndrome (yes)	0.99 (0.49; 1.98)
Diagnostic procedures during the episode	
X-ray	1.14 (0.94; 1.37)
MRI	0.88 (0.73; 1.05)
Other	1.00 (0.74; 1.35)
Imaging findings	
Disc degeneration	0.70 (0.54; 0.90)
Facet joint degeneration	1.02 (0.78; 1.33)
Scoliosis	1.00 (0.71; 1.42)
Spondylolisis	1.03 (0.59; 1.80)
Spondylolisthesis	0.86 (0.59; 1.25)
Spinal stenosis	0.75 (0.56; 1.02)
Disc protrusion or herniation (extrusion)	0.75 (0.61; 0.93)
Other non-relevant findings	0.97 (0.78; 1.19)
No findings	0.65 (0.47; 0.89)
Treatments	
Drugs	
Analgesics	0.89 (0.73; 1.09)
NSAIDs	1.26 (1.03; 1.54)
Steroids	0.80 (0.59; 1.08)
Muscle relaxants	1.11 (0.91; 1.36)
Opioids	0.78 (0.53; 1.14)
Other	0.87 (0.72; 1.05)
Non pharmacological treatments	
Physical therapy/Rehabilitation	1.00 (0.79; 1.28)
Years of experience	0.99 (0.98; 1.00)

* Radicular pain caused by disc protrusion/herniation or spinal stenosis.

**Table 8:** Multilevel full model to determine the strength of the association between predictors and improvement in disability (*n* = 4061).

In-Patient Improvement in Disability	Full Model
	OR (95% CI)
Predictor	
**Patient level**	
Gender (male)	0.96 (0.83; 1.11)
Age (years)	1.00 (0.99; 1.00)
Reason for referral to NRT	
Neck Pain	Ref. cat.
Low Back Pain	1.24 (1.00; 1.54)
Type of pain	
Non-specific	Ref. cat.
Radicular pain *	0.92 (0.67; 1.28)
Employment status	
Not working	Ref. cat.
Currently working	1.07 (0.90; 1.26)
Duration of the pain since diagnostic categorized	
≤1 year	Ref. cat.
1–5 years	0.79 (0.64; 0.99)
5–10 years	0.82 (0.65; 1.03)
>10 years	0.81 (0.64; 1.02)
Duration of the pain episode (days) categorized	
Subacute (≤90 days)	Ref. cat.
Chronic (91–365 days)	0.68 (0.58; 0.81)
Highly chronic (>365 days)	0.64 (0.53; 0.78)
Diagnosis of fibromyalgia	1.05 (0.72; 1.53)
Other comorbidities	0.86 (0.71; 1.03)
Involved in work-related claims	0.52 (0.25; 1.09)
Baseline severity of SP (VAS)	0.99 (0.96; 1.04)
Baseline severity of RP (VAS)	0.95 (0.93; 0.98)
Baseline disability (standardized 0–100 score)	1.00 (0.99; 1.01)
Previous lumbar surgery (yes)	0.61 (0.48; 0.77)
Failed back syndrome (yes)	0.42 (0.23; 0.79)
Diagnostic procedures during the episode	
X-ray	1.10 (0.93; 1.30)
MRI	1.02 (0.86; 1.20)
Other ¤	0.93 (0.71; 1.23)
Imaging findings	
Disc degeneration	0.80 (0.64; 1.00)
Facet joint degeneration	1.00 (0.79; 1.26)
Scoliosis	1.00 (0.74; 1.34)
Spondylolisis	1.16 (0.71; 1.91)
Spondylolisthesis	0.77 (0.55; 1.08)
Spinal stenosis	0.82 (0.61; 1.10)
Disc protrusion or herniation (extrusion)	0.91 (0.75; 1.10)
Other non-relevant findings	0.92 (0.76; 1.13)
No findings	0.97 (0.74; 1.29)
Treatments	
Drugs	
Analgesics	0.94 (0.78; 1.12)
NSAIDs	1.12 (0.94; 1.33)
Steroids	0.88 (0.66; 1.18)
Muscle relaxants	1.15 (0.96; 1.38)
Opioids	0.69 (0.49; 0.97)
Other	0.91 (0.76; 1.09)
Non pharmacological treatments	
Physical therapy/Rehabilitation	0.84 (0.67; 1.04)
Years of experience	0.99 (0.98; 0.99)

* Radicular pain caused by disc protrusion/herniation or spinal stenosis.

**Table 9:** Proportion of patients who improved and did not improve in the empty and full models.

	Empty Model		Full Model	
	Improved	Did Not Improve	Improved	Did Not Improve
Spinal pain	6169 (75.5%)	2001 (24.5%)	3548 (74.0%)	1243 (26.0%)
Referred pain	4343 (72.0%)	1681 (28.0%)	2530 (70.2%)	1076 (29.8%)
Disability	3673 (65.6%)	1925 (34.4%)	2661 (65.5%)	1400 (34.5%)

## Data Availability

The data analyzed and presented in this study are available upon request from the authors.
